# Personalized Approach to Patient with MRI Brain Changes after SARS-CoV-2 Infection

**DOI:** 10.3390/jpm11060442

**Published:** 2021-05-21

**Authors:** Ljiljana Marcic, Marino Marcic, Sanja Lovric Kojundzic, Barbara Marcic, Vesna Capkun, Katarina Vukojevic

**Affiliations:** 1 Department of Radiology, Polyclinic Medicol, Šoltanska 1, 21000 Split, Croatia; 2 Department of Neurology, University Hospital Center Split, Spinčićeva 1, 21000 Split, Croatia; marino.marcic@yahoo.com; 3 Department of Radiology, University Hospital Center Split, Spinčićeva 1, 21000 Split, Croatia; lovric.sanja@gmail.com; 4 Department of Medical Genetics, University of Mostar School of Medicine, Petra Krešimira IV bb, 88000 Mostar, Bosnia and Herzegovina; barbara.marcic@mef.sum.ba; 5 Department of Anatomy, Histology and Embryology, University of Split School of Medicine, Šoltanska 2, 21000 Split, Croatia; vesna.capkun@mefst.hr

**Keywords:** SARS-CoV-2, brain, MRI, neurological symptoms

## Abstract

From the beginning of SARS-CoV-2 virus pandemic, it was clear that respiratory symptoms are often accompanied with neurological symptoms. Neurological manifestations can occur even after mild forms of respiratory disease, and neurological symptoms are very often associated with worsening of the patient’s condition. The aim of this study was to show abnormal brain neuroimaging findings evaluated by MRI in patients after SARS-CoV-2 infection and neurological symptoms. Methods: Sixteen patients after mild forms of SARS-CoV-2 infection, twenty-three patients after moderate forms of SARS-CoV-2 infection as well as sixteen healthy participants in the control group underwent MRI 3T brain scan. All subjects in the SARS-CoV-2 group had small, punctuate, strategically located and newly formed hyperintense lesions on T2 and FLAIR sequences. New lesions were formed more often in the bilateral frontal subcortical and bilateral periventricular, correlated with the severity of the clinical picture. These changes indicate an example of silent cerebrovascular disease related to SARS-CoV-2 and once again emphasize the neurotropism of the virus.

## 1. Introduction

With the outbreak of the SARS-CoV-2 virus in 2020 and the development of a global pandemic, over 130 M infected patients have been detected worldwide, and over 2.9 M people have died. Central nervous system involvement is not unusual in patients with severe acute respiratory syndrome [1]. As the number of infected people increased, it was becoming increasingly clear that SARS-CoV-2 virus infection was a multisystem disease with very different clinical presentations [2]. Neurological complications were not very common in patients with SARS-CoV-2 at first, but increasingly recognized as the pandemic developed. Several mechanisms may be involved in the pathophysiology of the virus as well as damage to the nervous system, but full mechanisms are still not fully understood. Neurological manifestations can be caused by non-specific complications of systemic infectious disease, inflammation of the nervous system or dysfunction of cerebral blood vessels [3,4]. Direct viral invasion of the neural and supporting glial cells through ACE2 receptors, which are expressed in many parts of the brain, can be a basic pathophysiological mechanism [5]. Vascular endothelial inflammation and SARS-CoV-2 complement-induced coagulopathy cause diffuse endothelial dysfunction, impaired vasoreactivity, in most severe cases associated with thrombus formation in the microcirculation [6,7]. Another aspect of the pathophysiological mechanism is hyperactivity of the host immune system and molecular mimicry, which may further aggravate brain damage and clinical situation [8]. Autoantibodies against heat-shock proteins can also be involved because these antibodies are non-organ specific and are often found in the blood of patients with SARS-CoV-2 infection [9]. Prolonged hypoxia and electrolyte changes as a consequence of long-term respiratory disease may also contribute to the development of neurological complications.

The severity of the disease is divided into several groups: asymptomatic, mild, moderate, severe and critical. Asymptomatic patients had no clinical symptoms, had a positive nasopharyngeal swab and had a regular lung X-ray. Patients with a mild form of the disease had fever, cough, malaise, body aches, vomiting, abdominal pain and nausea. Patients with moderate disease had pneumonia (persistent fever and cough) without hypoxemia and with evident lesions on CT of the lungs. Patients with the severe form of the disease had pneumonia with hypoxemia. Critically ill patients had respiratory distress syndrome, coagulation disorder, encephalopathy or renal failure [10].

The evidence suggests that SARS-CoV-2 can result in a number of neurological symptoms and incidents, including smell and taste dysfunction, headache, dizziness, depression, anosmia, encephalitis, stroke, epileptic seizures and Guillain–Barre syndrome, among many others [11]. Central nervous system involvement is most commonly associated with more severe forms of the disease and a poorer prognosis, but even patients with mild respiratory symptoms may have neurological symptoms.

In patients who had a severe SARS-CoV-2 infection, the most common neuroimaging findings was the involvement of medial temporal lobe, non-confluent numerous hyperintensive lesions of the white matter in the FLAIR sequence showing different postcontrast imbibition, hemorrhagic lesions and diffuse microbleeds within the white matter in SWI sequence [12]. Anzalone et al. showed in four patients with severe SARS-CoV-2 infection and with neurological symptoms numerous laminar cortical lesions, which do not meet the criteria for neuroradiological entities but may indicate dysregulation and vasomotor reactivity by type of vasoconstriction [13]. Parsons et al. showed acute disseminated encephalomyelitis (ADEM) in patients with severe SARS-CoV-2 infection presented on neuroimaging as numerous periventricular and juxtacortical hyperintensive lesions in the FLAIR sequence increased intensity in DWI [14]. Hayashi et al. presented a patient with neurological symptomatology to whom MRI of the brain showed abnormal hyperintensity in the area of the corpus callosum with an increased signal in DWI and was suspected of encephalitis/encephalopathy [15]. Toledano-Massiah et al. showed two patients with a severe SARS-CoV-2 infection who had an MRI of the brain with unusual nodular and annular lesions periventricularly, and within the white matter, it was hyperintensive on the FLAIR sequence. The corpus callosum was also affected [16]. Flores et al. showed extensive pontine and midbrain hemorrhage with penetration into the 3rd and 4th ventricles [17]. Yiping et al. showed MRI volumetric and diffusion measurements of structural brain damage in patients who have recovered after SARS-CoV-2 infection [18].

The aim of this study is to draw attention to the newly formed brain changes that can be manifested by magnetic resonance imaging in patients who have overcome a mild and moderate form of viral infection. Finally, we will highlight linking the site of origin of new lesions with SARS-CoV-2 infection.

## 2. Materials and Methods

### 2.1. Population Study

By searching the database of the University Hospital Center of Split, we found 879 patients who were examined for SARS-CoV-2 infection in the emergency services during the period between 1 January and 31 March 2021. The first inclusion criterion was the severity of the clinical picture. Patients were classified as mild, moderate or severe based on WHO criteria [19,20]. There were 249 patients with a mild clinical picture, 510 with a moderate clinical picture and 120 with a severe clinical picture. In this clinical study, we excluded patients with a severe clinical picture, because such patients were not suitable for brain MRI in outpatient settings. In the group of patients who had a mild SARS-CoV-2 infection, 73 of them had nonspecific neurological symptoms (headaches, vertigo, impaired sense of smell and taste). In the group of patients who had a moderate SARS-CoV-2 infection, 206 of them had nonspecific neurological symptoms (headaches, vertigo, impaired sense of smell and taste). In the group of patients with a mild clinical picture, 23 of them were between the ages of 35 and 40, 22 patients were without comorbidities and risk factors, and 17 of them had a previous 3T brain MRI. In this group, one patient did not want to participate in the study, leaving 16 patients in the study. In the group of patients with a moderate clinical picture, 101 of them were between the ages of 35 and 40, 97 patients were without comorbidities and risk factors, and 32 of them had a previous 3T brain MR. In this group, 9 patients did not want to participate in the study, leaving 23 patients in the study. We found a control group of subjects between voluntary blood donors and postgraduate students by random screening between the ages of 35 and 40 years. We matched them according to age, gender and risk factors for cerebrovascular disease.

### 2.2. Methods

**Figure 1:**
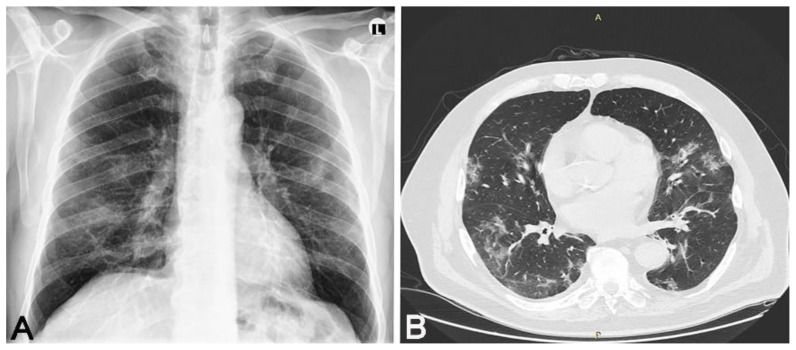
Thirty-eight-year-old male patient without any chronic diseases was admitted to the emergency department as a SARS-CoV-2 positive with high fever, cough, dyspnea and fatigue. Chest X-ray showed (**A**) bilaterally gentle inhomogeneous shading initial infiltrative changes. After 9 days from the onset of infection, dyspnea worsened, chest MSCT showed (**B**) areas of enhanced attenuation by the type of ground glass, and patchy consolidations are seen on both sides. The patient developed bilateral pneumonia. Legend: L—left side, small A letter on panel B: A—anterior, P—posterior.

**Figure 2:**
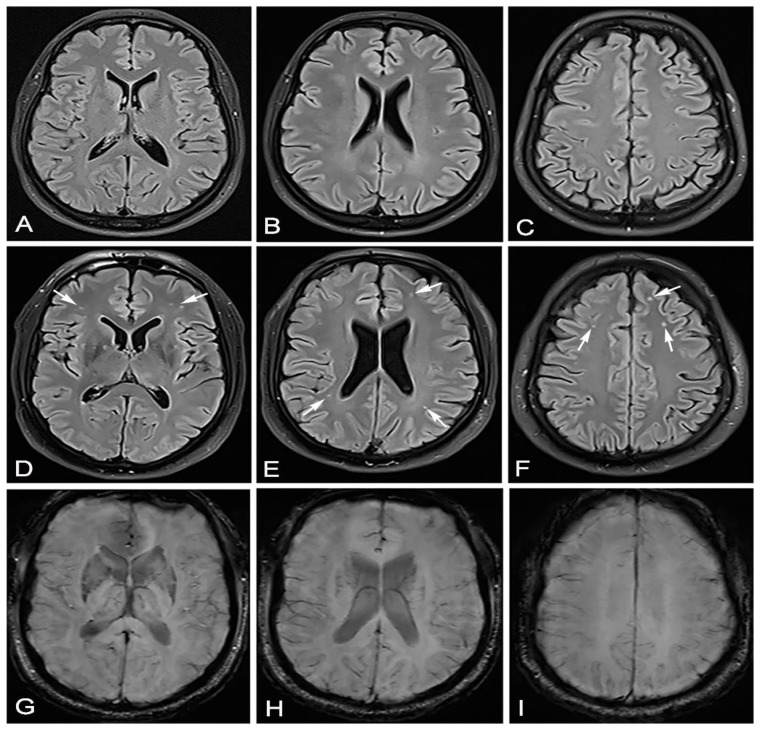
In June 2020, this male patient—as in Figure 1—had a completely normal brain MRI after vertigo (**A**–**C**). In March 2021, 53 days after recovering from moderate SARS-CoV-2 infection, the same patient came for a brain MRI due to headache, vertigo and taste and smell disorder. Brain MRI showed hyperintensive lesions on the FLAIR sequence located mostly bilaterally frontally subcortically and bilaterally periventricularly (**D**–**F**) (white arrows). The same transverse cross sections of the FLAIR sequence as on (**A**–**C**), without signs of microhemorrhage on the SWI sequence (**G**–**I**).

**Figure 3:**
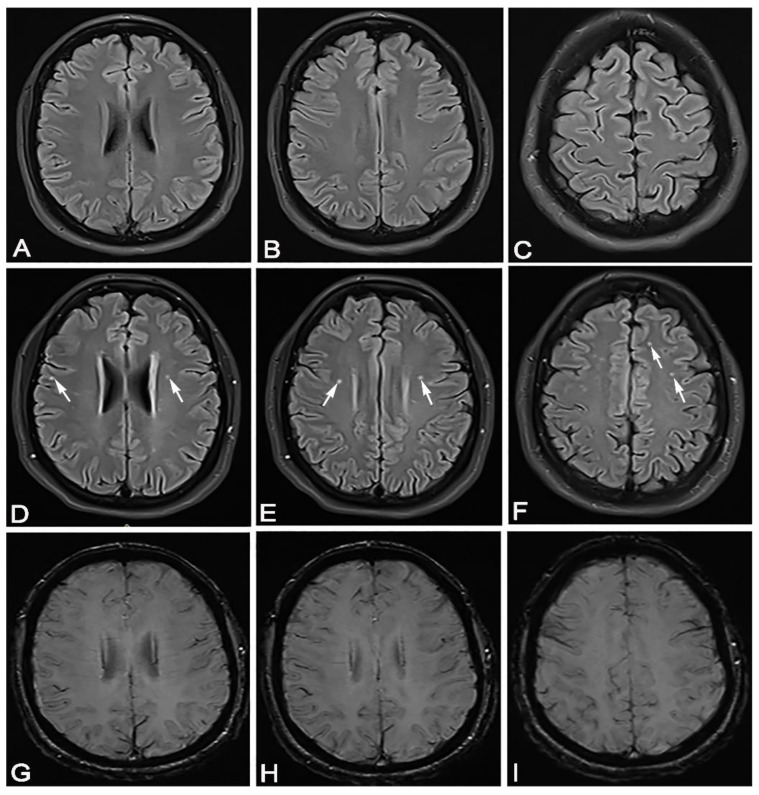
In April 2020, a 35-year-old female patient came for a brain MRI due to vertigo. (**A**–**C**) She showed completely normal brain MRI. In February 2021, 50 days after recovering from mild SARS-CoV-2 infection, the same patient came for a brain MRI due to headache, vertigo and taste and smell disorder. (**D**–**F**) She showed hyperintense lesions on FLAIR sequence, periventricular bilaterally, bifrontal subcortical and right juxtacortical (white arrows). The same transverse cross sections of the FLAIR sequence as on (**A**–**C**), without signs of microhemorrhage on the SWI sequence (**G**–**I**).

We conducted a cross-sectional observational study. We received informed consent from each participant. This study was conducted after obtaining permission from the local ethics committee. We had two groups of participants who overcame the SARS-CoV-2 infection; the diagnosis was confirmed by a positive result of a real-time reverse PCR test by nasal/pharyngeal swabs. In the first group of 16 participants, there were patients who overcame a mild form of the disease, and in the second group, there were 23 patients who overcame a moderate form of the disease. All patients who had a mild form of the disease had a normal X-ray of the lung, and patients with a moderate form of the disease had unilateral or bilateral pneumonia proven by MSCT. The group that overcame unilateral or bilateral pneumonia (Figure 1, Figure 2 and Figure 3) treated with antibiotic therapy, but without corticosteroid therapy, differed from the second group of participants, who had a mild form of disease.

All included 39 patients had nonspecific neurological symptoms such as smell and taste dysfunction, vertigo, headache, dizziness or fatigue. All subjects overcame SARS-CoV-2 infection from 40 to 60 days before MRI brain scan. The control group was 16 healthy volunteers (voluntary blood donors) who had no symptoms of SARS-CoV-2 infection and who were seronegative for SARS-CoV-2 IgG and IgM antibodies. All subjects were included from 1 January to 31 March 2021. All participants were Caucasian adults, aged 35–40 years. Participants’ data included age, gender, height, weight, body mass index, history of smoking, history of alcohol drinking, regular drug use, hypertension, diabetes, hyperlipidemia, many laboratory blood findings, coronary heart disease, atrial fibrillation and prior cerebrovascular disease. None of the included patients who had SARS-CoV-2 infection received corticosteroid therapy. We excluded all patients with a history of uncontrolled hypertension, unregulated diabetes mellitus, cerebrovascular disease, hematologic disease, atrial fibrillation, chronic heart disease or cancer, severe alcohol consumption (more than 10 drinks per week), known occlusive disease of cerebral arteries, stenosis of the vertebral artery or internal and external carotid artery more than 20%. We excluded all patients using anticoagulant or vasodilator drugs, hormone replacement therapy, β-blocking agents and calcium channel blockers. We excluded also patients who had severe neurological changes: stroke, epi seizures, Guillain–Barre syndrome, encephalitis (Table 1). All patients underwent ultrasound examination of the blood vessels of the neck (ACI, ACE, VA) using Siemens Acuson NX3 Ultrasound system (9.0 MHz frequency transducer) the same day they did the brain MRI. All included patients had a normal brain MRI scan before SARS-CoV-2 infection (6–12 month ago).

We used the VAS scale to assess the strength of the headache. Visual analogue scales (VAS) are psychometric response scales used to measure subjective pain. The visual analog scale (VAS) was used most frequently, with a total of 167 publications identified, in our research characteristics or attitudes of pain. The VAS consists of a 10 cm long horizontal line with its extremes marked as “no pain” and “worst pain imaginable”. In VAS, 1–3 points is equal to mild pain and has a minimal impact on activities of daily living (ADLs), 4–6 is equal to moderate pain with a moderate impact on ADLs, and 7–10 point is equal to severe pain with a major impact on ADLs. The visual analog scale (VAS) was the most frequently used scale in studies and clinical practice [21].

#### MRI Procedure

Brain MRI scans of the included patients was performed at 3 Tesla Skyra MRI scanner (Siemens Healthcare). We did all standard and additional sequences: T1-weighted imaging (axial, sagittal), fluid-attenuated inversion recovery—FLAIR (axial), T2-weighted imaging (axial, coronar), T2* susceptibility-weighted imaging (or T2*-weighted gradient-recalled echo if susceptibility-weighted imaging is not available), diffusion-weighted imaging with both a trace image and an apparent diffusion coefficient map (axial), CISS and with T1-weighted imaging enhancement. All included patients receiving paramagnetic contrast agent intravenously (gadoterate meglumine, dose 0.2 mL/kg). The T1 sequence was made from the postcontrast sequences. The diffusion-weighted trace image and apparent diffusion coefficient map are important for excluding recent infarcts. Sequence parameters should be consistent with recommendations for neuroimaging from the American College of Radiologists [20]. All MRI parameters for brain imaging are shown in Table 2. Additionally, we did MR angiography. All brain MRI findings were interpreted by two board-certified independent neuroradiologists (L.M., S.L.K).

### 2.3. Statistical Analysis

Statistical analysis was done with SPSS 20. Statistical significance was set to *p* < 0.05, and all confidence intervals were given at the 95% level. For numeric variables, the Shapiro–Wilk test was used to indicate deviation from normal distribution. Numeric variables were presented by median (Q1–Q3; min–max) or by mean ± SD. The statistical significance of the differences of categorical variables was calculated from the Fisher’s exact test and by binary logistic regression. Analysis of differences of numeric variables between two groups was done by Mann–Whitney U test analysis of differences of numeric variables between groups was done by the Kruskal–Wallis test.

## 3. Results

The research included 55 subjects. Of the total, 16 had a mild SARS-CoV-2 infection, 23 had a moderate form, and 16 subjects belonged to the control group, without SARS-CoV-2 infection. The age of the subjects was 35–40 years. All subjects underwent brain MRI 40–60 days after recovery from SARS-CoV-2 infection. In the control group, none of the subjects had diabetes mellitus or smoked; none had arterial hypertension and atrial fibrillation. In the group of subjects with a mild form of SARS-CoV-2 infection, there was one smoker and one with diabetes mellitus. In the group of subjects with moderate SARS-CoV-2 infection, there were three smokers, one with atrial fibrillation and three with arterial hypertension. Table 3 shows demographic data and laboratory findings in relation to the examined groups.

There is a statistically significant difference in hematocrit values between the examined groups (χ^2^ = 9.9; *p* = 0.007). The difference is made between the group with a moderate form of the disease, according to the group with a mild form of the disease and the control group. The difference between the median group with a moderate form of the disease and the group with a mild form of the disease is 0.003, and the difference between the group with a moderate form of the disease and the control group is 0.025, which we consider clinically irrelevant.

Erythrocyte values differed statistically significantly between the examined groups (χ^2^ = 18.8; *p* > 0.001). The difference is the group with a moderate form of the disease, according to the control group and the group with a medium form of disease, according to the group with a mild form of the disease. The difference between the median erythrocytes of the groups with moderate and mild forms of the disease is 0.2, and the difference between the group with a moderate form of the disease and the control group is 0.5, which we consider clinically irrelevant. All subjects were within normal limits.

There is a statistically significant difference in the BMI of the subjects in relation to the examined groups (χ^2^ = 25; *p* < 0.001). The median BMI in the group of subjects with a moderate form of SARS-CoV-2 infection was 3.7 (95% CI: 2.2–5.1), which is higher than in those with a mild form of SARS-CoV-2 infection and 3.4 (95% CI: 2–4.8) higher than in the control group.

There is a statistically significant difference in platelet counts, according to the examined groups (χ^2^ = 35.9; *p* < 0.001). The median platelet count in the group of patients with moderate SARS-CoV-2 infection was 321 (95% CI: 269–372), which is higher than in the control group. The median platelet count in the mild group was 194 (95% CI: 154–233), which is higher than in the control group. We did not prove a statistically significant difference in platelet counts between mild and moderate patients (*p* = 0.066) at a significance level of 95%. The median difference in platelet values between mild and severe patients was 127.

Subjects were divided according to platelets into two groups: 150–424 (within normal limits) and > 424 (Table 4).

In the group of subjects with moderate disease, there is a higher prevalence of increased platelet counts (>424) (*p* = 0.006). The probability of an increased platelet count is 8.6 times higher in the group of moderately ill patients than in the group with a mild form of the disease. The symptoms of the disease are shown in relation to the severity of the disease (Table 5).

We did not prove a statistically significant difference in the number of subjects with olfactory and taste impairments between the examined groups (*p* = 1). In the group with mild disease, all subjects had vertigo, while in the group of subjects with moderate disease, there were 11 subjects who suffered from vertigo (50%) (*p* < 0.001). There was a statistically significant difference in the degree of headache in relation to the severity of the disease (*p* < 0.001). In the group of subjects with a mild form of the disease, none of the subjects had a VAS of 5–10, but all 16 subjects had a VAS of 1–4. In the group of subjects with severe disease, 16 (69%) had VAS 5–7, and seven (31%) had VAS 1–4.

Patients with moderate SARS-CoV-2 infection had a statistically significantly higher IgG antibody level (z = 3.0; *p* = 0.002). The median IgG for 19 (95% CI: 11–27) is higher in those who presented a moderate form of the disease.

Patients with moderate SARS-CoV-2 infection had a statistically significantly higher number of brain lesions (z = 5.3; *p* < 0.001). The median number of lesions by 12.5 (95% CI: 9.7–15.3) is higher in the moderate form of the disease (Table 6).

**Figure 4:**
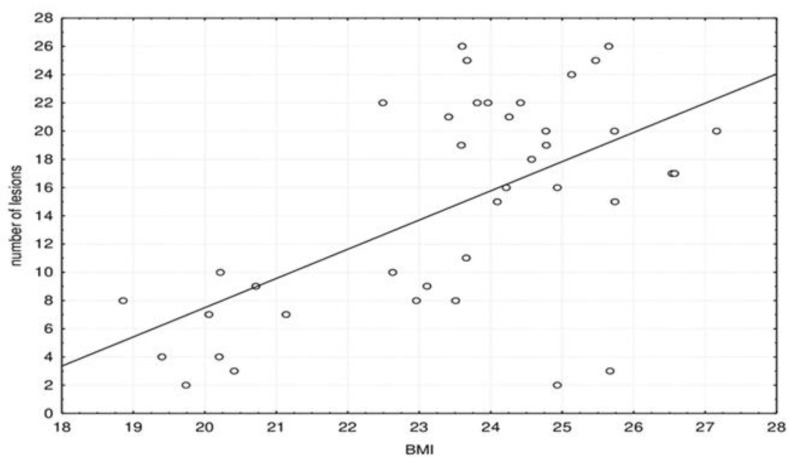
Scatter plot of the correlation between the number of brain lesions and BMI.

Furthermore, we investigated dependence of the number of lesions with symptoms, platelets and BMI. Figure 4 shows scatter plot of the correlation between the number of brain lesions and BMI.

The number of brain lesions significantly correlated with BMI: Spearman correlation coefficient rho = 0.469 (*p* = 0.003).

**Figure 5:**
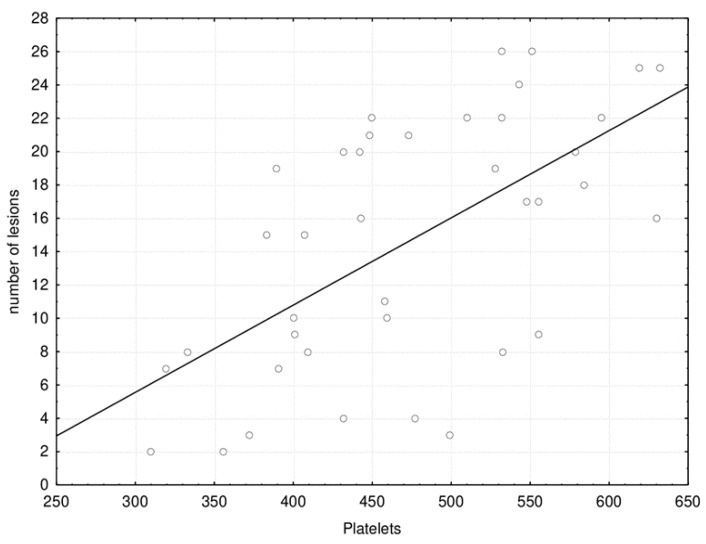
Scatter plot of the correlation between the number of brain lesions with platelet count.

Figure 5 shows the correlation between the number of brain lesions with the platelet count.

The number of brain lesions significantly correlated with the platelet count: Spearman correlation coefficient rho = 0.599 (*p* < 0.001).

**Figure 6:**
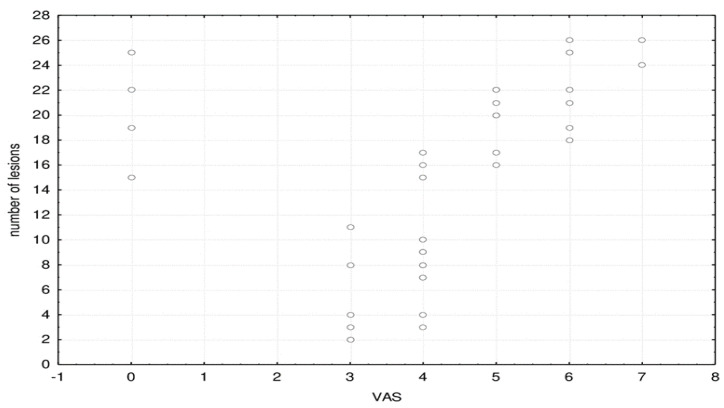
Scatter plot of the correlation between the number of brain lesions with the degree of VAS.

Figure 6 shows the correlation between the number of brain lesions with the degree of VAS.

The number of brain lesions significantly correlated with the VAS degree of headache: Spearman correlation coefficient rho = 0.578 (*p* < 0.001).

We did not prove an association between the number of lesions and damage of smell and taste (z = 0.928; *p* = 0.353). We did not prove an association between the number of lesions and vertigo (z = 1.92; *p* = 0.055) (Table 7).

At all three locations, there is a higher number of lesions in the moderate form of the disease. The median number of lesions is higher in moderate disease on the bilateral periventricular for three (z = 5.0; *p* < 0.001); it is higher by 10 on the bilateral frontal subcortical (z = 5.3; *p* < 0.001) and by one on bilateral frontoparietal juxtacortical (z = 2.4; *p* = 0.016) (Table 8).

## 4. Discussions

We presented patients after SARS-CoV-2 infection who developed nonspecific neurological symptoms as a headache, vertigo, dizziness and fatigue with multiple punctate T2/FLAIR hyperintensities on brain MRI. The lesions were distributed frontal subcortical bilaterally, periventricular bilaterally and juxtacortical bilaterally. The lesions did not show diffusion restriction, did not show a sign of hemorrhage on SWI, and also did not show postcontrast T1 enhancement. Additionally, the DWI sequence showed no signs of ischemia or infarction in any of the patients. CISS sequence showed perfectly normal vestibulocochlear nerve bilaterally. MR angiography was normal with no sign of vasculitis, and also, the previous MR angiography of the brain was normal. Additionally, all patients had a normal Doppler ultrasound of extracranial arteries of the neck the same date we did an MRI brain scan. So, we defined these lesions as a very small vascular lesion that occurred after SARS-CoV-2 infection, and they were not present on past MRI brain scans in 2019 and 2020. We also have to say that the reason, and the only symptom why these patients came to previous MRI brain scans, was vertigo.

Conklin et al. showed 16 severely ill patients with SARS-CoV-2 infection who had microvascular changes on brain MRI, which presented as >10 punctiform abnormalities in the SWI. The lesions had a predilection site subcortically and in deep white matter. They also showed radiological–pathological correlates of these microlesions, which included red blood cell extravasation, ischemia, deficiency, but not loss of axons and myelin, and microglial response [19]. Our patients did not have lesions in the corpus callosum, which may be a sign of a bad clinical picture and a worse clinical outcome [16,19]. Significant consequences and pathological changes after infection remained on all organs, especially on the lung parenchyma [22]. Brain changes cannot only be irreversible, but they are also cumulative over time. This brings us to the state that the brain is the organ that recovers the slowest after SARS-CoV-2 infection and on which the most lasting consequences remain. Additionally, our patients were younger, without arterial hypertension, amyloid angiopathy and multiple sclerosis. It is possible that our study involved patients who have initial endothelial changes and who require constant clinical and radiological monitoring in the following years, which ultimately poses a major public health problem. Patients who have overcome a moderate form of SARS-CoV-2 infection have a significantly higher total number of lesions. According to the Fazekas scale, the described lesions in patients with mild and moderate disease were grade 1. Although patients with moderate disease had a higher number of lesions, they were not confluent and were not larger than 10 mm. All described lesions were 2–3 mm in diameter [23]. We did not prove an association between the number of lesions and damage of smell and taste. We did not prove an association between the number of lesions and vertigo.

The location of the lesion is also evident in patients with mild and moderate forms of the disease in three locations: bilateral periventricular, bilateral frontal subcortical and bilateral frontoparietal juxtacortical. In both groups of patients, the lesions are statistically significantly grouped mostly bilaterally frontally subcortically, then bilaterally periventricularly. There was a statistically significant difference in the number of lesions at both sites in patients who had overcome a moderate form of SARS-CoV-2 infection. We considered it very important to emphasize that in both groups of patients, a higher number of lesions was bilateral frontally subcortical than bilaterally periventricularly, and significantly more in patients who had a moderate form of the disease. (Figure 3). SARS-CoV-2 shows similarities with SARS-CoV, which enters the CNS, as evidenced by preclinical and postmortem studies [24]. In our study, in both groups of patients, most of the lesions were located bilaterally frontally subcortically, and in the postmortem study, a virus was detected inside the frontal lobe [25]. The lesions were located mostly within the white matter and did not show the character of demyelination, although in their study, Zanin et al. showed patients with numerous confluent periventricular demyelinating lesions [26]. Therefore, this points to differences in brain MRI findings.

It is also important to note that the control group had a normal MRI brain scan and a normal Doppler ultrasound of the large blood vessels of the neck. In healthy people of this age who were shown in our study, we do not expect any brain lesions. There is a statistically significant difference in platelet counts, according to the examined groups. Platelet levels were slightly elevated in both groups of patients with SARS-CoV-2 infection. Patients who overcame the moderate form of SARS-CoV-2 infection had a slightly higher platelet count compared to the group of patients with a mild clinical picture. Existing studies showed thrombocytopenia as a post-SARS-CoV-2 complication [27]. We assume that our findings in this study cannot be ignored, and such patients should be further monitored due to the possible later development of a risk of thromboembolism and possible treatment with antiplatelet therapy. We found no elevated d-dimers in both groups of patients.

There is a statistically significant difference in the BMI of the subjects in relation to the examined groups. In the group of subjects with a moderate form of SARS-CoV-2 infection, BMI was higher than in those with a mild form of the SARS-CoV-2 infection and higher than in the control group. Obese patients have an increased risk of death from SARS-CoV-2 infection [28]. The association between elevated BMI and SARS-CoV-2 infection has biological and pathophysiological support. These include specific mechanisms such as chronic pro-inflammatory condition, excessive oxidative stress response and impaired immunity [29]. Elevated BMI and obesity are associated with a poorer clinical outcome [30]. Obesity has been recognized as a compromising factor of normal respiratory function and may place these patients in the group more severely affected by SARS-CoV-2 infection [31].

Patients with moderate SARS-CoV-2 infection had a statistically significantly higher IgG antibody level. Ren et al. have shown that antibody levels determine the severity of the disease [32]. Pradenas et al. showed that antibody titer was higher in patients who had a more severe form of SARS-CoV-2 infection than in those who had a mild/moderate form of the disease [33]. Patients with a more severe form of SARS-CoV-2 infection seroconvert earlier and had higher levels of IgG-specific antibodies than patients who had a mild form of the disease [34].

We did not prove a statistically significant difference in the number of subjects with olfactory and taste impairments between the examined groups. In the group of mild disease, all subjects had vertigo, while in the group of subjects with moderate disease, 11 subjects suffered from vertigo. There is a statistically significant difference in the degree of headache in relation to the severity of the disease. In the group of subjects with a mild form of the disease, none of the subjects had VAS of 5–10, but all 16 subjects had VAS 1–4. In the group of subjects with moderate disease, 16 had VAS 5–7, and seven had VAS 1–4. This means that the more severe the clinical picture, the more intense and pronounced the headaches were and points us to headache as a common and important nonspecific neurological symptom.

MRI is certainly the method of choice [35] for such patients who do not have expressed post-SARS-CoV-2 complications and difficulties, which are not dependent on other people’s help and are suitable for monitoring in outpatient institutions. MSCT is a method that is mostly insufficient and does not provide enough information in neuroimaging in such patients MRI brain (summary) radiopedia [36].

In 2003, still after SARS, we realized what post-infectious conditions can occur [37,38]. Neuropathological conditions are typically presented over a certain period of latency following an infectious illness. Today’s scientific knowledge considered that SARS CoV-2 virus affects the microcirculation, causing endothelial cell swelling and damage (endotheliitis), microscopic blood clots (microthrombosis), capillary congestion, pericytes damage, tissue repair (angiogenesis) and scar formation [39]. Similar to other instances of critical illness, SARS-CoV-2 is also associated with elevated cytokine levels in the systemic circulation [40]. The underlying mechanisms, which include penetration of the virus into the central nervous system are yet to be elucidated, and a direct correlation with SARS-CoV-2 infection remains uncertain, but we tried to make some conclusions.

Different mechanisms of cerebrovascular disorders might be involved in SARS-CoV-2. In a later phase, microthrombosis indicates small cerebral vessel involvement [41]. Central nervous system disorders associated with SARS-CoV-2 may lead to long-term disabilities [3]. Mechanisms should be urgently investigated to develop neuroprotective strategies.

We consider this study an example of silent cerebrovascular disease related to SARS-CoV-2, characterized by vascular damage predominantly involving microvessels [42]. Risk factors for silent CVD have been known for many years such as atherosclerosis, diabetes, hyperlipidemia, stress and aging [43]. Considering MRI brain findings today, the question arises as to whether SARS-CoV-2 is also a new risks factor for cerebrovascular disease. If this proves to be true, these patients require further monitoring and ongoing therapy, especially antiplatelet therapy [44], to prevent further unwanted cerebrovascular incidents.

Cerebrovascular diseases are the second leading cause of death in the world. Some classic risk factors have been known for years, such as obesity, hyperlipidemia, hypertension, diabetes, cigarette smoking, contamination and alcohol consumption, but new risk factors such as infectious agents have recently been documented. Chronic infections, such as chlamydia pneumoniae infection, human cytomegalovirus, helicobacter pylori infection, influenza virus and hepatitis C virus, can contribute to the development of atherosclerosis [45].

It is already known that the prevention of cerebral insult could be by three cardinal manifestations of silent cerebrovascular disease: silent cerebral stroke, magnetic resonance imaging white matter hyperintensities of presumed vascular origin and cerebral microbleeds [42].

This study leaves a number of questions that we will not be able to answer immediately. Monitoring of patients included in this study in future years, we will be able to elucidate the possible pathophysiological mechanisms involved in the processes within the CNS. Are they perhaps predisposed to CVI, dementia and vasculitis? Are such patients predisposed to other lung, heart or kidney diseases? Time will clear up all the consequences we will be able to evaluate.

Therefore, at this point, definitive understanding of disease mechanisms and causal relationships are limited. Furthermore, the underlying pathophysiologic processes may be concurrent and related, rather than distinct and independent; for example, a marked inflammatory response may be the cause of the observed cerebrovascular disease.

These changes indicated an example of silent cerebrovascular disease related to SARS-CoV-2, characterized by vascular damage predominantly involving microvessels and also once again emphasizes the neurotropism of the virus.

Following our study, it is obvious that the approach to patients after SARS-CoV-2 infection must be strictly personalized. In practice, this individual approach means finding patients with potential cerebrovascular disease, cognitive disorders and gait disorders. Location of the lesions in the frontal lobe must direct us to possible changes in the patient’s behavior after SARS-CoV-2 infection and that aspect of the disease should also be considered. This personalized approach to the patient will benefit the community and the global health system. This can lead us to create guidelines for neuroprotective therapy, help treat strategies for severe forms of the disease using ventilation or corticosteroid therapy, find biomarkers for the disease, improve outcomes and shorten treatment lengths. Ultimately, this will reduce the cost to the health system, which will allow the treatment of more people.

In practice, we can recommend that emergency departments in the questionnaires for patients on arrival must have data on BMI and quantified headache strength (VAS). Brain MRI imaging must be monitored at least over 6 to 12 months. TCCD should be included in the SARS-CoV-2 test algorithm and cerebral vasoreactivity measured. The cognitive status of these patients (MOCA questionnaire, evoked potentials) over a longer period should also be also assessed. All of this leads from a personalized approach to comprehensive care after a SARS-CoV-2 infection.

Conclusions: Every day during this pandemic, the SARS-CoV-2 virus has given us a new unpleasant surprise. This study showed that even patients who have mild respiratory symptoms may be burdened with neurological complications. In our case, it is a silent cerebrovascular disease that carries significant consequences for both the patient and the health system. The changes we found on MRI brain scan are a direct consequence of SARS-CoV-2 infection and reaffirm the multisystem nature of SARS-CoV-2 infection. Considering that these patients are young, without risk factors, it is concluded that SARS-CoV-2 infection is a new risk factor for the development of stroke. With the increased number of patients in the world, the conclusion is that soon post-SARS-CoV-2 cerebrovascular disease will become a significant public health problem. There is a question of monitoring such diseases as well as adequate treatment. We need further studies to shed light on this problem.

## Figures and Tables

**Table 1:** Inclusion and exclusion criteria.

Inclusion Criteria	Exclusion Criteria
Age from 35 to 40 years	Age under 35 and over 40 years
Mild form of respiratory SARS-CoV-2 disease treated exclusively with supportive therapy	Severe/critical form of SARS-CoV-2 pulmonary infection
Moderate form of respiratory SARS SARS-CoV-2 disease, pneumonia treated with antibiotic and supportive therapy	Disturbance in consciousness, acute cerebrovascular disease (ischemic stroke, cerebral hemorrhage, subarachnoid hemorrhage), acute encephalopathy, encephalitis or meningitis, polyneuropathy, demyelinating spectrum of disease and seizures.
SARS-CoV-2 infection from 40 to 60 days before MRI brain scan recording	More than 60 days from infection start and MRI brain scan
	Use of corticosteroids, oxygen for SARS-CoV-2 infection
A diagnosis confirmed by a positive result of real-time reverse PCR test by nasal/pharyngeal swabs	history of uncontrolled hypertension, unregulated diabetes mellitus, cerebrovascular disease, hematologic disease, atrial fibrillation, chronic heart disease or cancer
6–12 months earlier done brain MRI	Severe alcohol consumption (more than 10 drinks per week)
Nonspecific neurological symptoms such as smell and taste dysfunction, vertigo, headache, dizziness or fatigue	Stenosis of the extracranial vertebrobasilar artery > 20%
	Stenosis of the extracranial carotid artery > 20%.
For control group negative PCR SARS-CoV-2 test	Known occlusive disease of intracranial cerebral arteries
No SARS-CoV-2 symptoms at all	Use of anticoagulant drug, vasodilatory drugs, hormone replacement therapy, β-blocking agents, calcium channel blockers
	Severe neurological changes; stroke, epi seizures, Guillain–Barre syndrome, encephalitis.

**Table 2:** Brain MRI scan parameters.

	T1-Weighted	DWI	T2-Weighted	FLAIR	T2 Weighted SWI
TR (ms)	<650	4000	>2000	5000	25–50
TE (ms)	10–30	91	80–250	10–30	20–34
Flip angle (°)	90°	180°	90°	90°	5–30°
Matrix (pixel)	260 × 320	280 × 320	260 × 320	310 × 320	310 × 320
N° of slices (trans/sag)	44/30	44	44	44	44
Gap (mm)	0.8	0.8	0.8	0.8	0.8
FOV (mm)	280	280	280	280	280
Inversion time (ms)				1800–2200	

° degree.

**Table 3:** Display of the number of subjects (%) according to qualitative variables and median (min–max) or arithmetic mean ± SD of quantitative variables in relation to the examined groups (control group, groups with moderate and mild form of SARS-CoV-2 infection).

KERRYPNX	Groups of Subjects with SARS-CoV-2 Infection			
	Control Group	Mild Form	Moderate Form	*p*
Sex				
Male	10 (62.5)	10 (62.5)	13 (56)	0.905 †
Female	6 (37.5)	6 (37.5)	10 (44)	
Age (years)	37.5 (35–40)	38 (35–40)	38 (35–40)	0.718 *
BMI kg/m^2^	21.2 (19.4–25)	20.9 (19–26)	24.6 (22–27)	<0.001
Body temperature (°C)	36.5 (36–37.1)	36.5 (36–37)	36.6 (36–37)	0.791 *
RR systolic	123 ± 8.1	124 ± 8.2	127 ± 7	0.181 ††
RR diastolic	72.4 ± 5.9	75 ± 6.1	79 ± 6	0.003 ††
Hgb g/L	153 (137–165)	146.3 (125–162)	152 (134–160)	0.249 *
Hct L/L	0.525 (0.490–0.590)	0.497 (0.44–0.57)	0.50 (0.44–0.56)	0.007 *
Leukocytes × 10^9^/L	6.5 (4.8–8)	6.4 (4.9–9)	6.5 (4–9.2)	0.956 *
Platelets × 10^9^/L	211 (167–426)	405 (310–555)	532 (383–632)	<0.001 *
Erythrocytes × 10^12^/L	5.2 (4.9–5.9)	4.9 (4.4–4.7)	4,7 (4.1–5.5)	<0.001 *
Urea mm/L	4.9 (3.1–7.6)	5.5 (3.2–7.3)	5.0 (3.8–7.9)	0.557 *
Creatinine µm/L	72.5 (54–83)	65.5 (54–87)	78 (47–97)	0.138 *
Cholesterol mm/L	4.8 (4–5)	4.4 (4–5)	4.5 (4.1–5.4)	0.305 *
Triglycerides mm/L	1.3 (1.1–1.7)	1.5 (1.1–1.7)	1.6 (1.1–2)	0.077 *

* Kruskal–Wallis test; †† ANOVA; † χ^2^ test. †† There is a statistically significant difference (F = 6.7; *p* = 0.003) between values of RR days between the examined groups.

**Table 4:** Representation of the number (%) of subjects by groups (control, mild and moderate SARS-CoV-2 infection) in relation to normal (150–424) and elevated platelet counts (>424).

	Platelet Values × 10^9^/L		*p* *	OR (95% CI)	*p*
	150–424	>424			
Mild form (reference level)	9 (32)	7 (26)	0.006	8.6 (2–41)	0.007
Moderate form	3 (11)	20 (74)			
Control group	16 (57)	0			

* Fisher’s exact test. Note: the analysis in Table 2 refers only to the comparison of mild and moderate patients with SARS-CoV-2 infection.

**Table 5:** Display of the number (%) of subjects according to symptoms in relation to the severity of the disease.

		Degree of Disease		
		Mild	Moderate	*p* *
Impaired taste/smell	26 (67)	11 (69)	15 (65)	1.0
Vertigo	27 (69)	16 (100)	11 (48)	<0.001
Headache—VAS 1–4	23 (59)	16 (100)	7 (30)	<0.001
5–7	16 (41)	0	16 (70)	

* Fisher’s exact test.

**Table 6:** Median (IQR, min–max) IgG and number of lesions in relation to disease severity.

	Degree of the Disease		
	Mild	Moderate	*p* †
IgG U/mL	46 (38–55; 30–78)	65 (39–55; 38–89)	0.002
Number of lesions	7.5 (3–9; 2–11)	20 (17–22; 15–26)	<0.001

† Mann–Whitney U test.

**Table 7:** Display of the number of lesions in relation to olfactory damage and vertigo.

		Number of Lesions	
		Median (Q1–Q3; min–max)	*p* †
Impaired taste/smell	no	15.5 (3–21; 2–26)	0.353
	yes	16.5 (9–21; 2–26)	
Vertigo	no	18.5 (16–22; 15–26)	0.055
	yes	10 (7–21; 2–26)	

† Mann–Whitney U test.

**Table 8:** Display of median (min–max) number of lesions at three locations in relation to disease severity.

		Number of Lesions	
Location	Degree of the Disease	Median (Min–Max)	*p* †
Bilateral periventricular	mild	2 (0–3)	<0.001
	moderate	5 (3–9)	
Bilateral frontal subcortical	mild	4 (2–7)	<0.001
	moderate	14 (9–18)	
Bilateral frontoparietal juxtacortical	mild	1 (0–3)	0.016
	moderate	2 (0–5)	

† Mann–Whitney U test.

## Data Availability

Not applicable.
